# Prevalence of and risk factors for low bone mineral density in Spanish treated HIV-infected patients

**DOI:** 10.1371/journal.pone.0196201

**Published:** 2018-04-30

**Authors:** Miguel Cervero, Rafael Torres, Jose Luís Agud, Victoria Alcázar, Juan José Jusdado, Concepción García-Lacalle, Santiago Moreno

**Affiliations:** 1 Department of Internal Medicine, Severo Ochoa University Hospital, Leganés, Madrid, Spain; 2 Department of Endocrinology, Severo Ochoa University Hospital, Leganés, Madrid, Spain; 3 Department of Biochemistry, Severo Ochoa University Hospital, Leganés, Madrid, Spain; 4 Department of Infectious Diseases, Ramón y Cajal Hospital, University of Alcalá de Henares, IRYCIS, Madrid, Spain; Imperial College London, UNITED KINGDOM

## Abstract

**Objectives:**

Several studies have involved antiretroviral therapy in the pathogenesis of low bone mineral density (BMD), while others have not confirmed this association. In this study we analyze the impact of HIV status, traditional risk factors and antiretroviral therapy in BMD in an HIV-infected population living in Madrid.

**Material and methods:**

We performed a cross-sectional analysis of 107 individuals infected with HIV and exposed to antiretroviral treatment to estimate the prevalence of decreased BMD. Bone mineral density of lumbar spine and femoral neck was measured by dual-energy X-ray absorptiometry. In a multivariate analysis variables related with HIV status, antiretroviral drugs and traditional risk factors were included.

**Results:**

Low BMD was diagnosed in 63 participants (58.9%), including osteoporosis in 11 (10%). At least one cause of osteoporosis was identified in 43 patients (40%), with a deficiency of vitamin D in 86 (89%) and secondary hyperparathyroidism in 30 (28%). In multivariate analysis, increasing age, a treatment based on boosted PI and tenofovir DF, and previous exposure to tenofovir were identified as independent risk factors for a decreased BMD in both lumbar spine and femoral neck.

**Conclusions:**

We have confirmed a high prevalence of reduced BMD, which is favoured by ritonavir-boosted PI and TDF. Bone safety should continue to be evaluated in clinical trials and cohort studies in order to demonstrate that the new drugs offer additional advantages regarding the impact on BMD.

## Introduction

Life expectancy has increased significantly among people living with HIV since the introduction of combination antiretroviral therapy (cART)[1]. However, despite the improvement in their prognosis, patients living with HIV-infection, even under successful treatment, are at high risk of developing long-term complications. In particular, with an ageing population of HIV-infected patients, there is potentially an increased risk of low bone mineral density (BMD) secondary to the effects of chronic HIV infection and antiretroviral therapy [2].

Indeed, decreased BMD is a growing concern for HIV-infected individuals. Heterogeneous cross-sectional cohort studies, performed over the past ten years, have described a significantly higher prevalence of bone disease in HIV-positive individuals when compared to age-, race- and sex-matched HIV-negative controls [3]. A meta-analysis of pooled prevalence data from eleven cross-sectional studies performed between 2000 and 2005 demonstrated an overall prevalence of reduced BMD and osteoporosis of 67% and 15%, respectively, in 884 HIV-positive individuals [4]. When compared to 654 HIV-negative age and sex-matched controls, odds ratios were 6.4 (95% CI 3.7, 11.3) and 3.7 (95% CI 2.3, 5.9) for reduced BMD and osteoporosis, respectively. Also, there is evidence of low bone mass in HIV–infected young men on combination antiretroviral therapy (cART) [5], in both longitudinal and cross-sectional studies [6].

Hypothetically the prevalence of reduced BMD could be lower in countries with more hours of sunlight. The present study tries to estimate the prevalence of reduced BMD in a population-based cohort of Spanish HIV infected individuals receiving stable cART regimens. The enrolment was unrestricted including both genders and people of different sociodemographic characteristics. The study also investigated potential factors associated with decreased BMD and assessed whether cART itself was a risk factor.

## Material and methods

### Design and population of study

This cross-sectional study was carried out between January, 1985 and January, 2015 in a cohort of HIV-infected patients followed at Severo Ochoa University Hospital, in the southwest of Madrid (Leganés). Severo Ochoa University Hospital has a catchment of mostly a urban population of 180,000 inhabitants. The patients analyzed in this study are included in the COMESEM cohort, a larger cohort of HIV-1 infected patients followed at five different hospitals (metropolitan crown of southeastern of Madrid, including Leganés, Alcorcón, Getafe, Móstoles and Alcalá hospitals). It is an open and dynamic cohort with data collected both in a retrospective and prospective way.

The participants in the study are part of the COMESEM cohort. The organization and functioning of the cohort was approved by the clinical research and ethics committee of Alcalá on behalf of the rest of the hospitals (this is included in Spanish legislation) [7]. The clinical research and ethics committee approved the written informed consent of the patients. In Spain, clinical research and ethics committees evaluate studies with drugs.

The patients gave their informed consent to be included in the cohort and use their data for the purpose of research, not only for this study, without the need for additional consents. They were verbally informed of the information that was going to be obtained in the study’s analysis.

From the 450 patients of the COMESEM cohort followed in our hospital, 107 were included in this analysis. Inclusion criteria were being older than 18 years, receiving combination antiretroviral therapy, and having a bone densitometry performed during the period of follow-up. No selection criteria were applied to perform dual-energy X-ray absorptiometry (DXA). All the patients followed by the investigators had DXA routinely performed as part of their clinical care. Inclusion in the study was independent of treatment. No subject was on newer integrase inhibitors (elvitegravir or dolutegravir). Exclusion criteria were pregnancy, recent opportunistic infection, and current treatment with hormonal agents including testosterone, megestrol, or thyroid replacement therapy.

### Variables

Age, gender, clinical data and anthropometric measurements (including weight and height), as well as the history of HIV infection and ART were collected in each clinical visit. These data included risk practice for HIV acquisition, time of HIV infection, the lowest (nadir) and current CD4 cell count, current CD4:CD8 ratio, current and previous therapy, HIV RNA level and the composition and duration of current ART. In addition, individuals were asked about the presence of traditionally considered risk factors for reduced BMD (secondary causes of osteoporosis, smoking habits, alcohol consumption, methadone therapy, personal or familial history of osteoporosis or non traumatic fractures, corticosteroid therapy and diagnosis of autoimmune diseases). No patient was a current illicit drug injection user.

### Laboratory measurements

Blood samples were collected to analyze HIV related parameters (current CD4 cell count and current HIV viral load). As a general rule, blood samples were obtained within one month of DXA scanning. Glomerular filtration rate (GFR) was estimated by using the chronic kidney disease (CKD)-epidemiology collaboration equation. Also, individuals were screened for thyroid function (thyroid-stimulating hormone, TSH), hepatitis C (HCV-RNA), diabetes mellitus, gonadal function (total testosterone), vitamin D status (serum determination of 25-dihydroxyvitamin D by standardized electrochemiluminescence method (Cobas 601 Roche) and parathyroid function (serum parathyroid hormone, PTH) by chemiluminescence (Immulite 2000 Siemens).

The BMD was measured by dual X-ray absorptiometry (DXA) using Hologic densitometer (Hologic 4500, Bedford, USA) and included the values for bone mineral density (g/cm^2^), T-score or Z-score at level of L1-L4 lumbar spine and right or left femoral neck. The same DXA scanning was used throughout the study.

### Definitions

The use of T-score or Z-score for a diagnosis of low BMD depends of age of population of study. We used T-score when age was over 50 years, and it was defined as the number of standard deviations of difference in relation to the young adult population of the same gender. A diagnosis of osteoporosis or osteopenia was made when the score is –2.5 or between -2.5 and -1.0, respectively. < –1.0 was considered low BMD based on the World Health Organization criteria. We used Z-score if age was less than or equal to 50 years, and it was defined as the number of standard deviations of difference in relation to population matched according to age and gender. Scores of ≤ –2.0 standard deviations were considered to be low BMD based on World Health Organization criteria. Body mass index (BMI) was defined as the body mass divided by the square of the body height expressed in kg/m^2^, resulting from mass in kilograms and height in meters.

Risk factors for a decrease of BMD were considered according to the following definitions. Smoking was defined as current, active consumption. Alcohol intake was considered if there was daily consumption of three units or more (one unit = 10 g). Corticosteroid therapy was considered if taking at least 5 mg daily of prednisone during 3 months or more. Hepatitis C was defined in presence of a positive RNA-HCV by polymerase chain reaction. Diabetes mellitus was diagnosed if the patient had a previous diagnosis or if he was receiving glucose-lowering therapy. Hypogonadism was defined as testosterone deficiency in men, measured by a total testosterone level of less than 300 ng/dL. Vitamin D deficiency was defined as a value below 20 ng/ml. Secondary hyperparathyroidism was defined as an elevated plasma PTH above 65 pg/ml. CKD was established if estimated GFR (eGFR) values were lower than 60 ml/min/1.73 m^2^ (CKD stage 3 or higher). TSH levels below 0.50 mU/l defined hyperthyroidism.

### Statistical analysis

The study objective was to analyze the impact of HIV infection and the antiretroviral therapy in decreasing BMD adjusting by demographic, anthropometric and traditional risk factors. Description of variables was done showing frequencies and proportions for categorical variables, and calculating means, medians, and range for continuous variable. As previously stated, the prevalence of low BMD was calculated if T-score was < -1.0 or Z-score ≤ 2.0. Comparisons were performed by means of Student’s t or Mann–Whitney test for continuous variables, and chi-square test for categorical variables. A linear regression model was created with BMD value as the dependent variable, considered as continuous (g/cm^2^). We stratified multivariate analyses for lumbar spine and femoral neck BMD. Variables with p <0.30 in univariate analyses were included in the full model. Based on the plausible effect of TDF and PI/r on BMD, we have analyzed the impact of either drug alone (TDF, PI) or the combination of both. The final models were selected by using a stepwise descending procedure. Analyses were processed by the use of statistical package SPSS 20 for MAC. A *p* value less than 0.05 was considered statistically significant.

## Results

The study comprised 107 participants. They were mostly men (69.2%), and median age was 46.5 years [interquartile range (IQR), 43–51.5] (Tables 1, 2 and 3)).

**Table 1:** Baseline characteristics of the population of study: Sociodemographic characteristics.

VARIABLE*	
Sociodemographic characteristics	
Age (years)	46.5 (43–51.5)
Sex (men)	74 (69.2)
Risk category for HIV infection	
1.Intravenous drug use	46 (43)
2.Men who have sex with men	14 (13.1)
3.Sex among men and women	44 (41.1)
Race	
1.Caucasian	98 (91.6)
2.Non-Caucasian	9 (8.4)

* categorical variables are expressed as number of cases (percentage of the total); continuous variables are expressed as median (interquartile range), unless indicated otherwise.

**Table 2:** Baseline characteristics of the population of study: HIV-infection related characteristics.

VARIABLE*	
HIV-infection related characteristics	
CDC stage	
1. Stage A	46 (43)
2. Stage B	31 (29)
3. Stage C	30 (28)
Duration since HIV diagnosis (months)	188 (117–245)
Duration since ART initiation (months)	154 (69.5–187.5)
Number of regimens in the past	7 (4–11)
ART based on PI/r	53 (49.5)
ART based on TDF	63 (58.9)
ART based on non-TDF	44 (41.1)
ART based on PI/r & TDF	26 (24.3)
ART based on PI/r & no-TDF	35 (32.7)
Previous exposure to PI/r	90 (84.1)
Previous exposure to TDF	92 (86)
Duration of exposure to PI/r (months)	158.58 (58.08–276.12)
Duration of exposure to TDF (months)	119.42 (40.29–174.46)
Baseline CD4+ (cell/ μL)	251 (131.5–470)
Nadir CD4+ (cell/ μL)	127 (69.5–247)
Current CD4+ (cell/ mm^3^)	497 (345–705)
Baseline CD4:CD8 ratio	0.25 (0.13–0.52)
Current CD4:CD8 ratio	0.53 (0.34–0.84)
Baseline HIV viral load (RNA copies/mL)	100,000 (28,663–449,647)
HIV viral load <50 copies/mL	96 (89.7)

ART: antiretroviral therapy; CDC: Centers from Disease Control and Prevention; PI/r: ritonavir-boosted protease inhibitor; TDF: tenofovir disoproxil fumarate.

* categorical variables are expressed as number of cases (percentage of the total); continuous variables are expressed as median (interquartile range), unless indicated otherwise.

**Table 3:** Baseline characteristics of the population of study: Potential risk factors for low BMD.

VARIABLE*	
Potential risk factors for low BMD	
BMI (kg/m^2^), mean (SD)	25.03 (4.13)
Vitamin D < 20 ng/ml	86 (89.4)
PTH> 65 pg/mL	30 (28)
Active smoking	55 (51.4)
Consumption alcohol ≥ 3 unit	15 (14)
Methadone therapy	19 (17.8)
Hyperthyroidism (TSH < 0.5 mU/l.)	3 (2.8)
Corticosteroid therapy (Prednisone 5 mg ≥ 3 months)	None
Hepatitis C (HCV RNA)	43 (40.2)
Diabetes meliitus	7 (6.5)
Hypogonadism (testosterone < 300 ng/dL)	None
eGFR< 60 (ml/min/1.73m^2^)	4 (3.7)
Non-traumatic fractures	10 (9.3)
Numbers of factors	
1. 0 factor	8 (7.5)
2. 1 factor	43 (40.2)
3. 2 or more factors	56 (52.3)

BMI: body mass index; eGFR: estimated glomerular filtration rate; PTH: serum parathyroid hormone.

* categorical variables are expressed as number of cases (percentage of the total); continuous variables are expressed as median (interquartile range), unless indicated otherwise.

Median follow-up since the diagnosis of HIV infection was 188.5 months (IQR, 117–245). The most frequent risk group for HIV infection was former intravenous drug users (IDU)(43%), with 19 of them (17.8%) being on current methadone therapy. Thirty participants (28%) had developed AIDS. Median plasma viral load was <50 copies/ml in 89%. The median nadir and current CD4 cell count were 127 (IQR, 69.5–247) and 497 (IQR, 345–705) cells/μl, respectively. Chronic active hepatitis C (positive HCV RNA) was diagnosed in 40.2%. At the time of the study, all the participants were taking antiretroviral drugs. Regimens that patients were currently taking were as follows: TDF-based 58.9%; non-TDF based 41.1%. Among the non-TDF based regimens: 45.7% were not receiving any NRTI (including monotherapy with ritonavir-boosted PI in 19.6%), while 54.3% were receiving NRTIs other than TDF in combination with NNRTI (26.1%), a PI/r (17.4%), an integrase inhibitor (6.5%) or other NRTIs (4.3%). Overall, 49.5% were treated with PI/r. Regarding previous treatment, D4T had been used in 51.4% of the patients, with no differences between those currently on TDF/PI regimens (29%) and those on non-TDF/PI regimens (19%) (p = 0.235). The cumulative time on cART was 1283 patient-years.

Based on WHO criteria, low BMD was diagnosed in 63 participants (58.9%), including osteoporosis in 11 participants (10.3%). Secondary causes of low BMD were frequent. Forty-three participants (40.2%) had at least one cause of osteoporosis. We found vitamin D deficiency in 86 participants (89.4%) and secondary hyperparathyroidism in 30 (28%). Renal insufficiency (stage 3), diabetes mellitus and hyperthyroidism were less frequent. No participant was diagnosed of inflammatory disease nor was receiving steroids. Of note, among the traditional factors related to the low BMD, a history of non-traumatic fractures was detected in 10 participants (9.3%). We did not find a low BMI in our population with low BMD (average BMI 23.36 kg/m^2^).

In univariate analysis, age was associated with a reduced BMD regardless the site of involvement (femoral neck or lumbar spine). In addition, factors associated with a reduced BMD at the femoral neck included male gender, low BMI, active tobacco smoking, methadone therapy, chronic hepatitis C, prolonged time between HIV diagnosis and initiation of ART, previous exposure to PI/r, and number of causes of osteoporosis, while only the degree of immunosuppresion was associated with a reduced BMD at lumbar spine (Tables 4, 5, 6, 7, 8 and 9).

**Table 4:** Univariate analysis of risk factors associated with low BMD in lumbar spine: Sociodemographic characteristics.

VARIABLES	Low BMD n = 53 (49.5%)	Normal BMD n = 54 (50.5%)	*p*
Sociodemographic characteristics			
Age (years)	48.5 (45.50–52.50)	44.50 (41.50–48.75)	0.007*
Sex			
1.Men	40 (54.1%)	34 (45.9%)	0.161
2.Women	13 (39.4%)	20 (60.6%)	
Risk category for HIV infection			
1.Intravenous drug use	7 (50%)	7(50%)	0.212
2.Men who have sex with men	27 (58.7%)	19 (41.3%)	
3.Sex among men and women	19 (40.4%)	28 (59.6%)	
Race			
1.Caucasian	49 (50%)	49 (55.6%)	0.750
2.Non-Caucasian	4 (44.4%)	5 (50%)	

* p< 0.05.

**Table 5:** Univariate analysis of risk factors associated with low BMD in lumbar spine: HIV-infection-related characteristics.

VARIABLES	Low BMD n = 53 (49.5%)	Normal BMD n = 54 (50.5%)	*p*
HIV-infection-related characteristics			
CDC stage			
1. Stage A	28 (60.9%)	18 (39.1%)	0.086
2. Stage B	11 (35.5%)	20 (64.5%)	
3. Stage C	14 (46.7%)	16 (53.3%)	
Duration since HIV diagnosis (month)	194 (109.5–248.5)	185.5 (117–236.5)	0.423
Duration since ART initiation (month)	152 (71.5–178)	164.5 (57.25–189.5)	0.434
Number of regimens in the past	7 (4–13.25)	7 (4–11)	0.986
ART based on PI/r			
1.Yes	25 (47.2%)	28 (52.8%)	0.626
2.No	28 (51.9%)	26 (48.1%)	
ART based on TDF			
1.Yes	34 (54%)	9 (46%)	0.272
2.No	19 (43.2%)	25 (56.8%)	
ART based on PI/r & TDF			
1.Yes	16 (61.5%)	10 (38.5%)	0.159
2. No	37 (44.7%)	44 (45.3%)	
ART based on PI/r & no-TDF			
1.Yes	17 (48.6%)	18 (51.4%)	0.89
2.No	36 (50%)	36 (50%)	
Previous exposure of PI/r			
1.Yes	47 (52.2%)	43 (47.8%)	0.200
2.No	6 (35.3%)	11 (64.7%)	
Previous exposure of TDF			
1.Yes	46 (50%)	46 (50%)	0.811
2.No	7 (46.7%)	8 (53.3%)	
Duration of exposure on PI/r (months)	158.3(68.87–279.5)	161.37 (18.56–284.94)	0.896
Duration of exposure on TFV (month)	119(32.12–183.29)	123.62(40.31–165.1)	0.988
Baseline CD4 cell count (cell/ μL)	300 (158–474.5)	222 (110–455)	0.141
Nadir CD4 cell count (cell/ μL)	129 (75.5–250.5)	119 (46.75–249)	0.620
Current CD4 cell count (cell/ μL)	470 (286.5–663)	592(396–734)	0.025*
Baseline CD4:CD8 ratio	0.32 (0.16–0.54)	0.22 (0.08–0.54)	0.170
Current CD4:CD8 ratio	0.49 (0.32–0.81)	0.62 (0.38–0.93)	0.063
HIV viral load copies/mL	100000 (39810–566072)	79432 (25118–423877)	0.662
HIV viral load			
1.> 50 copies/mL	5 (45.5%	6 (54.5%)	0.775
2.< 50 copies/mL	48 (50%)	48 (50%)	

ART: antiretroviral therapy; CDC: Centers from Disease Control and Prevention; PI/r: ritonavir-boosted protease inhibitor; TDF: tenofovir disoproxil fumarate.

* p< 0.05.

**Table 6:** Univariate analysis of risk factors associated with low BMD in lumbar spine: Potential risk factors for low BMD.

VARIABLES	Low BMD n = 53 (49.5%)	Normal BMD n = 54 (50.5%)	*p*
Potential risk factors for low BMD			
BMI (kg/m^2^)	24.32 (4.72)	25.73 (3.36)	0.080
Vitamin D			
1.< 20 ng/ml	40 (46.5%)	46 (53.5%)	0.206
2.> 20 ng/ml	13 (61.9%)	8 (38.1%)	
PTHi			
1.> 65 pg/ml	19 (63.3%)	11 (36.7%)	0.075
2.< 65 pg/ml	34 (44.2%)	43 (55.8%)	
Active smoking			
1.Yes	31 (56.4%)	24 (43.6%)	0.146
2.No	22 (42.3%)	30 (57.7%)	
Consumption alcohol			
1.Yes	9(60%)	6 (40%)	0.382
2.No	44 (47.8%)	48 (52.2%)	
Methadone therapy			
1.Yes	10 (52.6%)	9 (47.4%)	0.766
2.No	43 (48.9%)	45 (51.1%)	
Hyperthyroidism (TSH < 0.5 mU/l.)			
1.Yes	1 (33.3%)	2 (66.7%)	0.569
2.No	52 (50%)	52 (50%)	
Hepatitis C (HCV RNA)			
1.Yes	25 (58.1%)	18 (41.9%)	0.144
2.No	28 (43.8%)	36 (56.2%)	
Diabetes			
1.Yes	2 (28.6%)	5 (71.4%)	0.251
2.No	51 (51%)	49 (49%)	
eGFR			
1.< 60 ml/min/1.73m^2^	2 (50%)	2 (50%)	0.985
2.> 60 ml/min/1.73m^2^	51 (49.5%)	52 (50.5%)	
Non-traumatic fractures			
1.Yes	5 (50%)	5 (50%)	0.975
2.No	48 (49.5%)	49 (50.5%)	
Number of factors			
1. 0 factor	3 (37.5%)	5 (62.5%)	0.746
2. 1 factor	21 (48.8%)	22 (51.2%)	
3. 2 o more factors	29 (51.8%)	27 (48.2%)	

BMI: body mass index; eGFR: estimated glomerular filtration rate; PTH: serum parathyroid hormone.

**Table 7:** Univariate analysis of risk factors associated with low BMD in femoral neck: Sociodemographic characteristics.

VARIABLES	Low BMD n = 50 (46.7%)	Normal BMD n = 57 (53.3%)	*p*
Sociodemographic characteristics			
Age (years)	49 (44.50–52.75)	44.50 (41.50–49)	0.004*
Sex			
1.Men	41 (55.4%)	33 (44.6%)	0.007*
2.Women	9 (27.3%)	24 (72.7%)	
Risk category for HIV infection			
1.Intravenous drug use	7 (50%)	7 (50%)	0.019*
2.Men who have sex with men	28 (60.9%)	18 (39.1%)	
3.Sex among men and women	15 (31.9%)	32 (68.1%)	
Race			
1.Caucasian	48 (22.2%)	50 (77.8%)	0.125
2.Non-Caucasian	2 (49%)	7 (51%)	

* p< 0.05.

**Table 8:** Univariate analysis of risk factors associated with low BMD in femoral neck: HIV-infected related characteristics.

VARIABLES	Low BMD n = 50 (46.7%)	Normal BMD n = 57 (53.3%	*p*
HIV-infection related characteristics			
CDC stage			
1. Stage A	19 (41.3%)	27 (58.7%)	0.228
2. Stage B	13 (41.9%)	18 (58.1%)	
3. Stage C	18 (60%)	12 (40%)	
Duration since HIV diagnosis (month)	229 (163.25–251.75)	165 (55.5–229)	0.006*
Duration since ART initiation (months)	167.5 (127.25–196)	121 (46.5–181.5)	0.012
Number of regimens in the past	8 (4–14)	6 (3–10.25)	1
ART based on PI/r			
1.Yes	23 (43.4%)	30 (56.6%)	0.494
2.No	27 (50%)	27 (50%)	
ART based on TDF			
1.Yes	34 (54%)	29 (46%)	0.272
2.No	19 (43.2%)	25 (56.8%)	
ART based on PI/r & TDF			
1.Yes	16 (61.5%)	10 (38.5%)	0.159
2.No	37 (44.7%)	44 (45.3%)	
ART based on PI/r & no-TDF			
1.Yes	15 (42.9%)	20 (57.1%)	0.576
2.No	35 (48.6%)	37 (51.4%)	
Previous exposure of PI/r			
1.Yes	46 (51.1%)	44 (38.9%)	0.037*
2.No	4 (23.5%)	13 (76.5%)	
Previous exposure of TDF			
1.Yes	43 (46.7%)	49 (53.3%)	0.996
2.No	7 (46.7%)	8 (53.3%)	
Duration of exposure on PI/r (months)	176.54(96.58–298.54)	121.33 (4.79–276.12)	0.111
Duration of exposure on TFV (month)	139.33(40.25–188.19)	117.42(30.62–163.17)	0.402
Baseline CD4 cell count (cell/ μL)	282 (139.25–470)	250 (118.5–473.5)	0.628
Nadir CD4 cell count (cell/ μL)	98.5 (49.75–246)	153 (83.5–263.5)	0.094
Current CD4 cell count (cell/ μL)	441.5 (294.75–662.5)	558(355.5–722.5)	0.157
Baseline CD4:CD8 ratio	0.23 (0.10–0.49)	0.29 (0.12–0.54)	0.745
Current CD4:CD8 ratio	0.48 (0.28–0.78)	0.58 (0.40–0.98)	0.030*
HIV viral load copies/mL	112946 (39810–845746)	79432 (25118–316227)	0.167
HIV viral load			
1.> 50 copies/mL	5 (45.5%	6 (54.5%)	0.929
2.< 50 copies/mL	45 (46.9%)	51 (53.1%)	

ART: antiretroviral therapy; CDC: Centers from Disease Control and Prevention;; PI/r: ritonavir-boosted protease inhibitor; TDF: tenofovir disoproxil fumarate.

* p< 0.05.

**Table 9:** Univariate analysis of risk factors associated with low BMD in femoral neck: Potential risk factors for low BMD.

VARIABLES	Low BMD n = 50 (46.7%)	Normal BMD n = 57 (53.3%)	*p*
Potential risk factors for low BMD			
BMI (kg/m^2^)	23.97 (4.37)	25.96 (3.70)	0.012*
Vitamin D			
1.< 20 ng/ml	40 (46.5%)	46 (53.5%)	0.927
2.> 20 ng/ml	10 (47.6%)	11 (52.4%)	
PTHi			
1.> 65 pg/ml	17 (56.7%)	13 (43.3%)	0.198
2.< 65 pg/ml	33 (42.9%)	44 (57.1%)	
Active smoking			
1.Yes	31 (56.4%)	24 (43.6%)	0.04*
2.No	19 (36.5%)	33 (63.5%)	
Consumption alcohol			
1.Yes	9 (60%)	6 (40%)	0.267
2.No	41 (44.6%)	51 (55.4%)	
Methadone therapy			
1.Yes	14 (73.7%)	5 (26.3%)	0.009*
2. No	36 (40.9%)	52 (59.1%)	
Hyperthyroidism (TSH < 0.5 mU/l.)			
1.Yes	1 (33.3%)	2 (66.7%)	0.637
2.No	49 (47.1%)	55 (52.9%)	
Hepatitis C (HCV RNA)			
1.Yes	25 (58.1%)	18 (41.9%)	0.049*
2.No	25 (39.1%)	39 (60.9%)	
Diabetes			
1.Yes 2.No	4 (57.1%) 46 (46%)	3 (42.9%) 54 (54%)	0.568
eGFR			
1.< 60 ml/min/1.73m^2^	3 (75%)	1 (25%)	0.248
2.> 60 ml/min/1.73m^2^	47 (45.6%)	56 (54.4%)	
Non-traumatic fractures			
1.Yes	5 (50%)	5 (50%)	0.828
2.No	45 (46.4%)	52 (53.6%)	
Number of factors			
1. 0 factors	2 (25%)	6 (75%)	0.044*
2. 1 factors	17 (39.5%)	26 (60.5%)	
3. 3 o more factors	31 (55.4%)	25 (44.6%)	

BMI: body mass index; eGFR: estimated glomerular filtration rate; PTH: serum parathyroid hormone; TDF: tenofovir disoproxil fumarate.

* p< 0.05.

In multivariate analysis, an increasing age (b-0.008, p 0.001), current ART based on PI/r and TDF (b-0.181, p 0.001 in lumbar spine and b-0.083, p = 0.038 in femoral neck) and having ever been exposed to TDF (b-0.108, p = 0.008 in lumbar spine and b-0.122, p = 0.004 in femoral neck) were independent risk factors for a decreased BMD in both lumbar spine and femoral neck. Additional independent risk factors were identified for each of the two sites. At lumbar spine, intravenous drug use as a risk practice for HIV infection (b+0,162, p = 0,001 and b+0.117 and p = 0.003 homosexual and heterosexual versus intravenous drug users, respectively) and consumption of ≥3 alcohol units (b-0.106, p = 0.008) were related to a decreased BMD, while at the femoral neck only a lower BMI (b-0.007, p = 0.037) was independently associated (Tables 10, 11, 12, 13, 14 and 15).

**Table 10:** Multivariate analysis of factors associated with decreasing BMD in lumbar spine: Sociodemographic characteristics.

VARIABLES	Unadjusted Coefficient	*p*	Adjusted Coefficient	*p*
Sociodemographic characteristics				
Age (years)	-0.003	0.168	-0.008	0.004*
Sex (Women ref.)	-0.029	0.390		
Risk category for HIV infection				
1.Men who have sex with men	0.104	0.034*	0.162	0.001*
2.Sex among men and women (Intravenous drug use ref)	0.075	0.024*	0.117	0.003*
Race (Non-Caucasian ref)	-0.030	0.597		

* p< 0.05.

**Table 11:** Multivariate analysis of factors associated with decreasing BMD in lumbar spine: HIV-infection related characteristics.

VARIABLES	Unadjusted Coefficient	*p*	Adjusted Coefficient	*p*
HIV-infection related characteristics				
Duration since HIV diagnosis(month)	0.0001	0.570		
Duration since ART initiation(month)	0.00003	0.767		
Number of regimen in the past	-0.001	0.856		
ART based on PI/r (No PI ref)	0.009	0.767		
ART based on TDF (No TDF ref)	-0.083	0.008*		
ART based on PI/r & TDF (No PI/r anTDF ref.)	-0.092	0.011*	-0.181	<0.001*
ART based on PI/r & no-TDF (No PI/r and no-TDF ref.)	0.055	0.101		
Previous exposure of PI/r (Never PI/r ref)	-0.027	0.533		
Previous exposure of TDF (Never TDF ref.)	-0.067	0.140	-0.108	0.008*
Duration of exposure on PI/r (month)	0.0001	0.962		
Duration of exposure on TDF (mont)	0.0001	0.105		
Baseline CD4 cell count (cell/ μL)	0.0001	0.365		
Nadir CD4 cell count (cell/ μL)	0.0001	0.434		
Current CD4 cell count	0.00002	0.427		
Baseline CD4:CD8 ratio	0.036	0.098		
Current CD4:CD8 ratio	0.036	0.098		
HIV viral load > 50 copies/ml (>50 copies/ml ref.)	0.008	0.882		
Baseline HIV viral load	0.0001	0.969		

ART: antiretroviral therapy; PI/r: ritonavir-boosted protease inhibitor; TDF: tenofovir disoproxil fumarate.

* p< 0.05.

**Table 12:** Multivariate analysis of factors associated with decreasing BMD in lumbar spine: Potential risk factors for low BMD.

VARIABLES	Unadjusted Coefficient	*p*	Adjusted Coefficient	*p*
Potential risk factors for low BMD				
BMI (kg/m^2^)	0.004	0.989		
Vitamin D (> 20 ng/ml ref.)	0.062	0.119		
PTHi (< 65 pg/ml ref.)	-0.082	0.018*		
Active smoking (No ref.)	-0.046	0.148		
Consumption alcohol (No ref.)	-0.102	0.023*	-0.106	0.008*
Methadone therapy (No ref.)	-0,035	0.396		
Hyperthyroidism (No ref.)	-0.069	0.474		
Hepatitis C (HCV RNA) (No ref.)	-0.071	0.025*		
Diabetes (No Diabetes ref.)	0.048	0.451		

BMI: body mass index; PTH: serum parathyroid hormone.

* p< 0.05.

**Table 13:** Multivariate analysis of factors associated with decreasing BMD in femoral neck: Sociodemographic characteristics.

VARIABLES	Unadjusted Coefficient	*p*	Adjusted Coefficient	*p*
Sociodemographic characteristics				
Age (years)	-0.005	0.003*	-0.008	<0.001*
Sex (Women ref.)	0.0001	0.97		
Risk category for HIV infection				
1.Men who have sex with men	0.062	0.198		
2.Sex among men and women (Intravenous drug use ref)	0.056	0.085		
Race (Non-Caucasian ref)	-0.090	0.102		

* p< 0.05.

**Table 14:** Multivariate analysis of factors associated with decreasing BMD in femoral neck: HIV-infection related characteristics.

VARIABLES	Unadjusted Coefficient	*p*	Adjusted Coefficient	*p*
HIV-infection relate characteristics				
Duration since HIV diagnosis(month)	0.0001	0.061		
Duration since ART initiation(month)	0.0001	0.169		
Number of regimens in the past	-0.003	0.263		
ART based on PI/r (No PI ref)	0.023	0.445		
ART based on TDF (No TDF ref)	-0.031	0.325		
ART based on PI/r & TDF (No PI/r and TDF ref.)	-0.015	0.188	-0.083	0.038*
ART based on PI/r & no-TDF (No PI/r and TDF ref.)	0.018	0.574		
Previous exposure of PI/r (Never PI/r ref)	-0.065	0.121		
Previous exposure of TDF (Never TDF ref.)	-0.063	0.149	-0.122	0.004*
Duration of exposure on PI/r (month)	0.0001	0.491		
Duration of exposure on TFV (mont)	0.0001	0.415		
Baseline CD4 cell count (cell/ μL)	0.0001	0.472		
Nadir CD4 cell count (cell/ μL)	0.0001	0.547		
Current CD4 cell count	0.000005	0.926		
Baseline CD4:CD8 ratio	-0.035	0.095		
Current CD4:CD8 ratio	-0.039	0.749		
HIV viral load (>50 copies/ml ref.)	-0.002	0.963		
Baseline HIV viral load	0.0001	0.969		

ART: antiretroviral therapy; PI/r: ritonavir-boosted protease inhibitor; TDF: tenofovir disoproxil fumarate.

* p< 0.05.

**Table 15:** Multivariate analysis of factors associated with decreasing BMD in femoral neck: Potential risk factors for low BMD.

VARIABLES	Unadjusted Coefficient	*p*	Adjusted Coefficient	*p*
Potential risk factors for low BMD				
BMI (kg/m^2^)	0.007	0.064	0.007	0.037*
Vitamin D (> 20 ng/ml ref.)	-0.055	0.134		
PTHi (< 65 pg/ml ref.)	-0.033	0.033*		
Active smoking (No ref.)	-0.077	0.011*		
Consumption alcohol (No ref.)	-0.083	0.057		
Methadone therapy (No ref.)	-0.040	0.320		
Hyperthyroidism (No ref.)	-0.095	0.305		
Hepatitis C (HCV RNA) (No ref.)	-0.042	0.017*		
Diabetes (No Diabetes ref.)	0.012	0.852		

BMI: body mass index; PTH: serum parathyroid hormone.

* p< 0.05.

## Discussion

In this cohort study performed in an HIV-infected population with a median age closed to 50 years, a reduced BMD indicative of osteopenia and/or osteoporosis was found in 58.9% of the patients. Predisposing factors of reduced BMD in this population have been identified, including some in common with the non-HIV infected population (age, male sex, a lower BMI, active tobacco use and methadone therapy) as well as some specific factors related with HIV-infection (low CD4 cell count, HCV co-infection, longer duration of HIV infection and of combination antiretroviral treatment, specially the duration of PI/r).

The prevalence of reduced BMD in our cohort is consistent with that found in other cross-sectional studies in sunny climates, carried out in southwestern France[8], other regions in Spain [9,10], and India[11]. It has been established that the HIV-infected population has a lower BMD when compared with the non-HIV infected population[4]. The underlying mechanism triggering bone mineral loss in individuals with HIV infection is unknown. It has been proposed that the abnormalities in bone and mineral metabolism may be caused by direct invasion of the cells of the bone and bone marrow microenvironment, chronic T-cell activation and abnormal cytokine production affecting osteoblast and osteoclastic functions, disturbances of calcium homeostasis, parathyroid hormone functions, vitamin D metabolism, opportunistic or neoplastic disease and adverse effects of drugs[12].

In general, in addition to being a direct consequence of the viral infection *per se*, the bone demineralization may be related with some classical host factors or be an effect of combination antiretroviral therapy. Several factors in HIV-infected patients that might be expected to result in decreased BMD have been identified. The association of increasing age with decreasing T-score value has been documented in the general population[13], and it has been confirmed in our study. A possible effect of the HIV transmission group has been observed. We have found a relationship between reduced BMD and intravenous illicit drug transmission as the mechanism of HIV infection, similar to other studies[6]. However, other authors have documented that the population of men who have sex with men (MSM) has reduced BMD[14]. In fact, a recent study has found a low BMD in the younger MSM subjects, both HIV-infected and HIV-uninfected[15]. In this particular study, a lower body weight largely explained the lower BMD in treated HIV-positive individuals.

Specifically considered risk factors for low BMD in the HIV population in previous studies are the start of ART, use of some antiretroviral drugs, CD4 cell count at the initiation of the treatment, inflammatory state and the concomitant presence of hepatitis B and/or C in addition to the traditional factors associated to the style of life as consumption of alcohol (≥3 units/day), active smoking, opiate use, low weight, hypogonadism, prolonged exposure to steroids, renal disease, liver cirrhosis, vitamin D deficiency, diabetes, hyperthyroidism, hyperparathyroidism, Caucasian race and sedentary lifestyle[1,16].

Vitamin D deficiency deserves special comments. Similar to other studies, we found a high prevalence of hypovitaminosis D (89%), despite the many sunlight hours per year in Madrid. In a review carried out in Spain, hypovitaminosis D was considered to be a consequence of low dietary intake and of interference with sun exposure [17]. In a previous study, our group showed the results of a nutritional and lifestyle survey, in which only participants with 67 minutes/daily of sun exposure had sufficient levels of vitamin D [18]. Of note, the high prevalence of deficit of vitamin D does not apparently match with the prevalence of secondary hyperparathyroidism we found (28%). Similar discrepancies have been shown by other authors[19]. A plausible explanation is that the cut-off level used to define hyperparathyroidism may underestimate its real prevalence. At this respect, it has been pointed out that PTH levels defining biochemical hyperparathyroidism should be reassessed as BMD significantly declines even at levels within the currently defined normal range [11].

The role of antiretroviral drugs in the development of reduced BMD is a matter of debate. While several studies have involved cART in the pathogenesis of low BMD, others have not confirmed this association[20–22]. Ritonavir-boosted PI and TDF have been the antiretroviral drugs more frequently associated with a lower BMD[23–27]. Our data in a Spanish population are consistent with these findings. In the multivariate analysis we found that the antiretroviral regimes based on PI/r and TDF, as well as the previous exposure to TDF were all associated with decreased BMD.

Although the multivariate analysis showed that the antiretroviral regimes based on PI/r and TDF, as well as the previous exposure to TDF, were all associated with decreased BMD, the magnitude of the reduction may not be clinically relevant. We used absolute BMD (continuous) rather than low BMD (binary) because it is the procedure most frequently used both in observational studies and clinical trials that explore the effect of drugs on BMD.

Boosted PI might be involved in bone metabolism by increasing osteoclast differentiation of circulating mononuclear cells and impairing vitamin D metabolism by inhibition of 25 alfa hydroxylation at the hepatocyte level and 1-alfa hydroxylation in the monocyte[2,12]. Tenofovir alters the transcriptional profile of osteoblasts, altering genes involved in cell signaling, cell cycle and amino acid metabolism and because it has the ability to induce isolated renal phosphate wasting[3]. There are data that suggest a synergistic effect of boosted PIs and TDF based on the interaction between RTV and TDF. RTV inhibits active TDF secretion by the proximal tubule, resulting in an increase of plasma TDF concentrations by 25–35%[28]. In addition, comparing with other drug classes, PIs are associated with decreased BMD regardless of NRTI backbone[25,29], suggesting that the mechanism of their effects go beyond alterations in TDF metabolism.

Clinical trials have suggested stabilization of loss of BMD 6 to 12 months after initiation of ART[23,25,30]. However, recent observational studies have found that HIV-infected individuals continue to lose BMD after the rapid decline during the first 2 years of ART, although at a slower rate[31]. If these data were confirmed they would strongly argue for antiretroviral drug selection with smaller bone toxicity.

The conclusions of our study may be limited by several arguments. Since this is a cross-sectional study, no causality can be established between risk factors and a low BMD. Multivariate analysis was unable to eliminate confusion variables related to the duration of the exposure to each factor. In addition the small sample size could limit the external validity of our data, and the cross sectional design does not allow to provide prospective data regarding BMD course and therefore it is not powered to demonstrate a greater rate of bone loss in HIV positive individuals.

We did not have direct comparison between those in the cohort with and without DXA. For that, we must include selection bias in the limitations.

In summary, the observation of a lower BMD in HIV infected individuals makes it necessary to select those regimes with a smaller impact in the bone health. Our data support that, considering aging of HIV population and their frequent toxic habits (tobacco, alcohol and opiates), the regimes based on PI/r and tenofovir can favour the decrease of BMD. Therefore, we should go on evaluating the bone safety in clinical trials and cohort studies to try to demonstrate that the new drugs offer additional advantages regarding BMD.
